# Coordinated Health in Texas Elementary Schools’ Campus Improvement Plans: Analysis of Regional Differences and Trends between 2016 and 2020

**DOI:** 10.3390/ijerph19094979

**Published:** 2022-04-20

**Authors:** Sarah Classen, Jacob Szeszulski, Nalini Ranjit, Genesis Rivas-Ponce, Deanna M. Hoelscher

**Affiliations:** 1 Michael & Susan Dell Center for Healthy Living, The University of Texas Health Science Center at Houston (UTHealth), Austin, TX 78701, USA; s.c.classen3@gmail.com (S.C.); jacob.szeszulski@agnet.tamu.edu (J.S.); nalini.ranjit@uth.tmc.edu (N.R.); genesisjrivas@gmail.com (G.R.-P.); 2 Center for Medical Ethics & Health Policy, Baylor College of Medicine, Houston, TX 77030, USA; 3 Institute for Advancing Health Through Agriculture, Texas A&M AgriLife Research Center, Dallas, TX 75252, USA

**Keywords:** policy, physical activity, nutrition, mental health, bullying, children and adolescents, schools, school improvement plans

## Abstract

Schools signal health priorities through policies. Using a repeated cross-sectional study design, we compare the presence and strength of policies related to four topics—physical activity, nutrition, mental health, and bullying—described in elementary school Campus Improvement Plans (CIPs; also called school improvement plans) within Texas, across four Texas Public Health Regions (PHRs), and between 2016 and 2020. CIPs were collected using a multi-stage probability-based survey approach, scored using an adapted WellSAT tool, and analyzed to determine associations between PHR or year and health topic. Across 170 CIPs, bullying was the most frequently addressed topic, followed by mental health, physical activity, and nutrition. On average, schools addressed 2.7 ± 1.3 topics within their CIP; 38.2% of schools addressed all four, 26.5% addressed three, 12.4% addressed two, 15.3% addressed one, and 7.6% addressed none. CIPs in the same district had high levels of clustering (ICCs = 0.28–0.55). The mostly rural Panhandle PHR included the fewest topics in their CIPs and used the weakest policy language. Between 2016 and 2020, there was a decrease in the proportion of CIPs that addressed nutrition; the strength of language for mental health and bullying also decreased. Regional and time trends reveal opportunities for more robust school health policy interventions.

## 1. Introduction

A substantial portion of children’s and adolescents’ waking hours are spent in the school setting [1,2]. Global agencies, including the United Nations and World Health Organization (WHO), tout the potential for school environments to positively impact students’ health and recommend that schools implement strategies that focus on multiple health outcomes (e.g., physical activity, nutrition, bullying, mental health). For example, the WHO’s “Health Promoting Schools’’ framework provides a comprehensive approach for schools to implement health promotion programming that improves physical activity and healthy eating, decreases bullying, and provides mental health support [3]. Further, the Center for Disease Control (CDC) and Prevention’s Whole School, Whole Community, Whole Child model and their push for Comprehensive School-based Physical Activity programs suggest that schools look beyond programs, policies, and practices that focus on a single health outcome, and instead promote more coordinated health approaches [4,5].

School districts prioritize coordinated health and, accordingly, their school health environments through policies that engage parents/guardians, campus educators, staff, and community leaders in the development process [6]. Additionally, the effective delivery of wellness initiatives requires strongly worded policies with corresponding processes for evaluating implementation [7,8,9]. In the United States, local wellness policies—required for school districts participating in the National School Lunch or Breakfast Programs—set district-level goals for improving nutrition and physical activity environments, which districts evaluate through a triennial assessment process [2,10,11]. Strongly worded local wellness policies coupled with active school wellness committees have effectively enforced wellness policies, improved activity and eating environments, and led to positive student body mass index and nutrition outcomes [12]. However, local wellness policies are not required to cover topics such as mental health or bullying. Further, local wellness policies, written at the district level, are not always effectively communicated, translated, and implemented at the school level, which suggests that the assessment of other policies may be needed to better understand schools’ health priorities [13].

At the national level, the Every Student Succeeds Act necessitates that state education agencies identify schools that need improvement and that these schools engage families and community members in identifying challenges and developing solutions that are formalized into school improvement plans [14]. In the state of Texas, all schools are required to share with the public their Campus Improvement Plans (CIPs; also called school improvement plans), which are school-level documents that set annual priorities for improving the school health environment. After Texas Senate Bill 892 was signed into law in 2009, it became mandatory for all elementary, middle, and high schools to establish goals in their CIP for the following components of the school environment: physical activity, academic progress, violence prevention, and coordinated health programs. It is also mandatory that Texas schools adopt a coordinated school health program curricula that focuses on physical activity, nutrition, and mental health [15]. CIPs, which often reference the coordinated health curricula, are also required by state law to be released annually [16]. Consequently, assessing change in school-level policies over time can provide a sense of school health needs, including physical activity and nutrition, identify progress in implementing coordinated health approaches from previous years, and set priorities going forward.

Regional variation in CIPs may also reflect health trends occurring in different parts of the state, as children’s health outcomes are known to have regional variation based on the social determinants of health (SDOH) or environmental differences in that area (e.g., food access). Regional differences in the SDOH are also related to school environments, assessing student needs, and providing access to resources [17]. For example, multiple studies show urban–rural differences in access to health resources and student health outcomes. Additionally, in the southwestern United States, Texas–Mexico border regions have their own set of challenges. For example, border communities have poverty and obesity rates that are higher than the national average and these associations are related to differences in the food environment [18]. Accordingly, it is important to understand school policy differences related to health environments in border and non-border regions.

Thus, the purpose of this study was three-fold. First, we examined a sample of Texas CIPs to identify the language involving physical activity, nutrition, bullying, and mental health goals. Second, we used a repeated cross-sectional study design to examine changes in CIP language over a four-year period. Finally, we stratified CIPs by region to explore regional differences in policy topics.

## 2. Materials and Methods

This study uses a repeated cross-sectional study design to compare the presence and written policy strength for selected health topics within CIPs in four of the Texas Department of State Health Services’ Public Health Regions (PHRs) [19] and changes over a four-year period (2016–2020). In the 2015–2016 and 2019–2020 school years, CIPs were identified and collected as part of the Texas School Physical Activity and Nutrition Survey (Texas SPAN), a statewide childhood and adolescent obesity surveillance project that monitors body mass index, nutrition, and physical activity trends among public school children in Texas.

### 2.1. Sampling Methods

**Figure 1:**
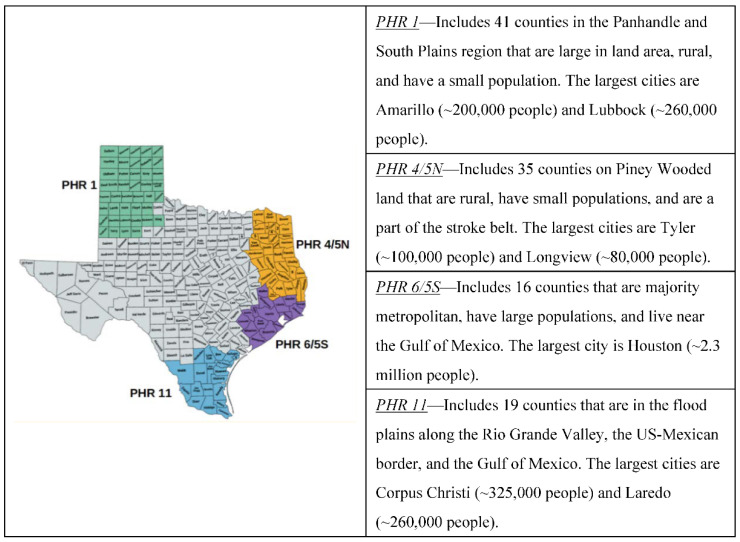
Texas Public Health Regions.

Sampling methods for this analysis are based on the larger Texas SPAN study [20]. Briefly, Texas SPAN is a multi-stage probability-based survey approach that intends to identify and measure the weight status and health behaviors of a sample representative of 2nd-, 4th-, 8th-, and 11th-grade youth in Texas at the PHR level and in border and non-border PHRs. To identify this representative sample, Texas SPAN collects information on and enrolls schools within Texas based on their demographic characteristics. Schools are stratified at the PHR level by their urban or rural status and their location in relation to the US–Mexico border (yes, no). Students are further stratified by their race/ethnicity (African American, Hispanic, or White/Other) and their grade (4th, 8th, or 11th). Based on stratifying characteristics, a random order to recruit school districts and schools is generated. If a school district or school declines participation, they are replaced by the next school district or school on the list. This process continues until the study is sufficiently powered (i.e., α = 0.05, power = 80%, and a detection limit of 3% for the state and 6.5% for each PHR, for a two-sided test) to estimate the prevalence of obesity for students at the PHR level and by each stratifying characteristic. Given the importance of developing health behaviors early in childhood and the limited sample size of schools at the middle and high school levels [21], we specifically focus on elementary schools that agreed to participate in the 2015–2016 and 2019–2020 Texas SPAN cycles. PHRs that were not geographically distinct or had insufficient sample sizes were also excluded. (i.e., PHRs 2/3, 7, 8, and 9/10). PHRs included in this analysis—Panhandle, East Texas, Major Metropolitan, US–Mexico border—are displayed in Figure 1.

### 2.2. Campus Improvement Plan Identification and Health Topic Selection

Once district and school approvals were obtained for the Texas SPAN study, trained project staff began the data collection process. For each school that agreed to participate in the larger Texas SPAN study, research staff obtained a copy of the school’s CIP from their online web page or requested it from school staff. For each CIP, the research staff examined the presence and policy strength for the following health variables: physical activity, nutrition, bullying, and mental health. Physical activity was selected as a topic given that Texas Senate Bill 892 specifically mentions “fitness” and “moderate-to-vigorous physical activity” as topics that should be addressed in the CIP [16]. We also selected nutrition as a topic because nutrition and physical activity are both included in districts’ wellness policies, nutrition is mandated as part of coordinated school health programs, and the Coordinated Approach to Child Health (CATCH)—an evidence-based physical activity and nutrition curriculum—is widely used in Texas school districts [2,22,23]. Bullying was selected as a topic area because Texas Bill 892 requires violence prevention to be included in CIPs, and the prevalence of bullying on school campuses is high–nearly a quarter (22.7%) of youth in the U.S. are bullied [16]. The negative health effects of being bullied include depression, anxiety, poor health, and suicidal behavior, which can persist into adulthood [24]. Furthermore, cyberbullying is an emerging health problem that schools are increasingly trying to prevent [25]. Finally, mental health was selected as a topic area because of its ties to bullying and heightened acknowledgement of mental health challenges among students amid the COVID-19 pandemic [26]. Together, these four topics—physical activity, nutrition, bullying, and mental health—provide a snapshot of school policy priorities within coordinated and comprehensive approaches to improving child health.

### 2.3. Measurement Methods

For each topic area, we created a list of key search terms used to ascertain if each topic was included in a school’s CIP. Two authors (DH, SC) generated an initial list of keywords for each topic area was generated by two authors (DH, SC) based on a review of the literature. Next, we reviewed materials on the Texas Education Agency’s (TEA) website to identify additional keywords. Materials include web pages, school resources guides, and school health surveys [27,28,29,30]. Finally, a team of content experts generated additional keywords and reviewed/approved the finalized list. Table 1 shows a finalized list of keywords generated through this process. When scoring the CIPs, we searched “General Terms” first and sorted them into the other four content areas, which were then subsequently scored with the other CIP language within that topic area.

We scored the presence (yes/no) and strength (range 0–3) of language for each topic area within the schools’ CIPs. To score the strength of language in each CIP, we adapted the WellSAT tool—a reliable and valid instrument for scoring local wellness policies [31]. However, unlike the WellSAT tool, strength scores were given per topic area (e.g., physical activity), instead of by policy item. Given that CIPs included very few sentences per topic area (typically one to two sentences), we used the highest rated policy item to calculate the score for the whole content area. A score of “0” was given when none of the keywords for that content area were found within the CIP. When a keyword was included but did not have a recommendation associated with the word, a score of “1” was given (e.g., Activity/Strategy: Implement Grandparent’s Week ***Lunch***). A score of “2” was given when a keyword was mentioned, but the recommendation was weak, and follow-through was determined to be difficult (e.g., Fun Fridays are for 5 min of extra ***recess***). Finally, a score of “3” was given when the keyword was included in a strongly worded recommendation (i.e., Goal 2: The District will promote a positive culture and climate that embraces the social and emotional needs of all students that reflects the District’s Core Values. Action 2.3.1: Promote an anti-***bullying*** campaign). To help differentiate between weak and strongly worded goals, we also examined other words within the policy recommendation. Weak language goals included words like “may”, “should”, “could”, and/or “can”. Strong language goals included words like “shall”, “must”, “require”, “enforce”, and “will” [31]. We also created two summary variables, one that aggregated the total number of topic areas included in a CIP and the other which scored the total strength of CIP language across all policy areas.

One researcher (SC) independently scored each CIP by searching for all the keywords and recording policy strength per topic area (four scores total per CIP) to complete the scoring process. When questions arose about whether the wording was strong or weak, the researcher flagged the policy item and reviewed it with another research team member (DH). The two team members (SC, DH) discussed the flagged item until consensus was reached. A different researcher (GRP) independently scored a subsample (~12%) of CIPs using the same process for data quality control. There was high inter-rater reliability between coders (90%).

### 2.4. Descriptive Characteristics

In addition to analyzing the CIPs, we used the TEA website to identify the schools’ descriptive characteristics, including the proportion of students of each race/ethnicity, the proportion of students who were economically disadvantaged, the proportion of students who were English language learners, and proportion of students in special education. We also collected information on the total number of students per school, campus expenditures per student, and school location (major urban, urban, or rural). Descriptive statistics were from the most recently available year (2019–2020).

### 2.5. Statistical Analysis

We used chi-squared tests, independent sample *t*-tests, and one-way ANOVAs to characterize schools based on their PHR and Texas SPAN cycle. Next, we identified the level of clustering (schools within districts) present for each topic area. Based on the high level of clustering for both the presence (e.g., presence of bullying) and strength of CIP scores within each district, we used general linear mixed-models that controlled for district-level clustering to determine both the unadjusted and adjusted associations between PHR or Texas SPAN cycle and each CIP outcome variable. Covariates included in the fully adjusted models were limited to the total number of students, campus expenditures per student, percentage of students who were economically disadvantaged, percentage of students who were English language learners, and PHR.

## 3. Results

### 3.1. Sample Description

We obtained 173 CIPS, 99 for 2015–2016 and 74 for 2019–2020. Three CIPs were excluded because the documents were incomplete (one from cycle 2015–2016 in PHR 6/5S and two from cycle 2019–2020 in PHRs 6/5S and 1). Thus, 170 CIPs were included in the repeated cross-sectional analysis (98 for 2015–2016 and 72 for 2019–2020). Seven schools were measured in both cycles, so we removed the older observation from the regional analysis leaving 163 CIPs for the final regional analysis (91 for 2015–2016 and 72 for 2019–2020). We surveyed fewer schools in 2019–2020 than in 2015–2016, as COVID-19 shifted organizational priorities and prevented us from completing data collection in that year. However, most PHRs contributed a similar number of CIPS across cycles, except PHR 6/5S, which contributed 36 CIPs in the 2015–2016 cycle and 14 CIPs in the 2019–2020 cycle. The average number of CIPs per school district across Texas SPAN cycles was 3.21 ± 3.56. Intra-class correlation (ICC) among schools in the same district was high for the presence of a topic and quality score for physical activity (ICC = 0.31 for presence; ICC = 0.47 for quality), nutrition (ICC = 0.55; ICC = 0.51), mental health (ICC = 0.28; ICC = 0.31), and bullying (ICC = 0.30; ICC = 0.33). As expected, due to the diversity of populations in different areas of Texas, there were statistically significant demographic differences between schools across PHRs for almost all variables (Table 2); however, only the proportion of black students and the total number of students per school demonstrated a significant decrease, whereas the proportion of special education students and campus expenditures per student significantly increased across Texas SPAN cycles.

### 3.2. Presence of Topic Areas Addressed within CIPs

Across all 170 CIPs pooled across the two years, bullying was the most frequently addressed health topic area (76.5%), followed by mental health (73.5%), physical activity (65.9%), and nutrition (56.5%). On average, schools addressed 2.7 ± 1.3 of four topic areas within their CIP; 38.2% of schools addressed all four areas, 26.5% addressed three topic areas, 12.4% addressed two topic areas, 15.3% addressed one topic area, and 7.6% addressed no topic areas. Unadjusted models examining changes across Texas SPAN cycles revealed that the proportion of schools that included physical activity, mental health, and bullying topics in their CIP did not change significantly over time; however, there was a statistically significant decrease in the proportion of CIPs that addressed nutrition (56.9% in 2015–2016 vs. 56.1% in 2019–2020; *p* = 0.038). Still, the total number of topic areas addressed was not significantly lower in the 2019–2020 cycle (M = 2.6; SD = 1.4 topic areas) compared to the 2015–2016 cycle (M = 2.8; SD = 1.2 topic areas; *p* = 0.448). Adjusted models did not change these findings. Across PHRs, CIPs in PHR 1 were least likely to address each of the four topic areas and, on average, addressed the fewest number of topic areas in total (Table 3).

### 3.3. Strength of Language within CIPs

Across all 170 CIPs, the strength of the language within the CIPs was strongest for bullying (M = 2.2; SD = 1.3), followed by mental health (M = 1.9; SD = 1.3), physical activity (M = 1.8; SD = 1.4), and nutrition (M = 1.2; SD = 1.3). On average, relative school policy strength across all four topic areas was 1.8 (SD = 1.0). Across Texas SPAN cycles, the strength of the policy language did not change for physical activity or nutrition, but the strength of CIP language for mental health (M = 2.0 SD = 1.3 vs. M = 1.7 SD = 1.4; *p* = 0.022) and bullying (M = 2.4 SD = 1.2 vs. M = 1.8 SD = 1.4; *p* = 0.018) was significantly higher in the 2015–2016 cycle compared to 2019–2020 cycle. Still, the average strength of language across all policy areas was not significantly higher between the 2015–2016 cycle (M = 1.9; SD = 0.9) and the 2019–2020 cycle (M = 1.6; SD = 1.0). Across PHRs, PHR 11 was significantly higher than PHR 1 in overall strength score and higher than PHR 4/5N in nutrition and bullying policy scores (Table 4). PHR 6/5S was also significantly higher than PHR 4/5N in bullying policy strength score. The adjusted models erased all findings related to bullying and revealed that PHR 6/5S was significantly lower (M = 1.3; SE = 0.2) than PHR 11 (M = 2.2; SE = 0.2) in physical activity policy strength score.

## 4. Discussion

We found a high level of clustering for all health policy-related outcomes within CIPs located in the same district. Although we expected some similarities between these policies, the high level of clustering indicates that CIPs may not be as locally tailored to the individual campus or school as we initially expected. Further, during the CIP review and scoring process, it became apparent that some schools use a district improvement plan as a template for their CIPs, and that schools within the same district utilize many of the same resources when writing a CIP [32,33]. Districts often use state-level templates when writing their local wellness policies, so it is unsurprising that schools also use templates when developing and writing their CIP [2,34]. The use of district-level templates and the high levels of clustering in this study suggest that policy intervention at the district level may have the most significant impact on changing school CIPs. However, implementation strategies that help schools better tailor CIPs to their local barriers, facilitators, and needs are also warranted [35,36].

Within CIPs, the most addressed topic areas were bullying and mental health; physical activity and nutrition were addressed less often. The finding that physical activity was addressed in only 65% of CIPs is surprising, as Texas state law specifically requires that CIPs include information related to physical activity, academic progress, violence prevention, and coordinated health programs within their CIPs [16]. However, physical activity is not specified as a component of district improvement plans [37]; thus, schools that rely on the district plan as a template may miss important topics that must be included in CIPs. Nutrition is not required to be included in CIPs, so it could have been anticipated that it was included in the fewest number of CIPs. However, despite the lack of a mandate for nutrition, over 50% of schools included it in their CIP, supporting our assertion that other policies may inform CIP content. Finally, the Texas education code does not require district or campus improvement plans to be submitted to a state agency for review, only that they are available upon reasonable request [37]. Numerous studies have found that implementation oversight and enforcement of health policies are related to higher quality health policies [38,39]. Thus, identifying mechanisms to review and enforce the CIP contents required by Texas state law may be one avenue to generating more comprehensive CIPs. Furthermore, it may be important to synergize the requirements of the district and campus-level improvement plans for better alignment and consistency.

Over the four-year period between the Texas SPAN cycles, there was a decrease in the proportion of CIPs that addressed nutrition as a topic area and decreases in the strength of CIP language for mental health and bullying; however, the total number of topics addressed and the overall strength of language within policies did not significantly change. Further, changes over time were not substantial. For example, there was less than a 1% change in the proportion of schools that included nutrition in their CIP. The average strength of policy language for mental health and bullying only decreased by about half a point. These findings are consistent with research on local wellness policies, which show relatively little change in strength and comprehensiveness scores between 2008 and 2014 [40]. Further, average strength scores ranged from one (topic included with no recommendation) to two (weak recommendation). Thus, interventions to improve the strength of language in CIPs are needed, as the strength of language within the policies is related to higher quality school health environments and better youth health outcomes [9].

Our exploratory regional analysis revealed that PHR 1, the rural Texas Panhandle region, scored significantly lower than PHR 11, the US–Mexico border region, for the total number of topics and strength of policy language in their CIPs. Further, rural PHR 4/5N had weaker nutrition and bullying language in their CIPs than more urban PHRs (65/S and 11). Although it is unclear from our analyses why differences exist between PHRs, we hypothesize that these differences may be due to the location of districts included in this study. In PHRs 1 and 4/5N, greater than 40% of the schools were rural, whereas, in PHRs 6/5S and 11, greater than 80% of the schools were in urban areas. Rural schools face additional barriers to developing and implementing health policies, including fewer staff that manage multiple job responsibilities, lack of technical and financial assistance, and challenges associated with the procurement of healthy foods [40,41,42]. It may also be that schools in PHR 1 have less or differing health needs than other PHRs, or there may be differences in funding for implementing school health programs. However, these hypotheses should be examined further.

### 4.1. Limitations and Strengths

This study includes several limitations; most of these relate to the incompleteness of data rather than bias. First, data collection for the 2019–2020 Texas SPAN cycle is lower than typical cycles due to the emergence of COVID-19 and school closures. Thus, CIPs for this cycle only include schools that were surveyed prior to school closures in mid-March 2020. Specifically, this affected PHR 6/5S as it contributed a smaller number of CIPs than it had in the previous cycle. Second, this report measured the presence and strength of variables in written policies but not the comprehensiveness of items within a policy topic (e.g., number of best practices for physical activity). Comprehensive policies are equally vital for changing school health environments and should be assessed in future CIP research [9]. Finally, several Texas PHRs were not included in this analysis due to inadequate data representation. These PHRs also have distinct environmental and geographic characteristics that may influence a CIP’s contents, and these associations should be explored in future studies. For example, during the process of reviewing CIPs, we noticed qualitative differences in the structure of district templates within and between PHRs (e.g., nearly half (43.75%) of PHR 1 CIPs were very short—less than 10 pages—for the 2019–2020 cycle). Conducting qualitative research to better understand CIP development and structural patterns should be considered in future studies.

One strength of this study is the large sample of geographically diverse CIPs that we obtained through a multi-stage probability-based survey approach. The use of a multi-stage probability-based survey approach allowed us to obtain a random sample of CIPs that were representative of various geographic PHRs, which facilitated comparisons of CIPs between regions and generated hypotheses about how the location of a CIP can affect its contents. Second, we adapted a validated tool to measure the strength of language within policies. This adapted approach allowed us to quickly generate scores for multiple content areas within a single policy, which is important when assessing comprehensive and coordinated approaches to school health programming. Finally, we collected policy data longitudinally. Currently, few studies collect health policy data beyond a single time point. By collecting data over a four-year period, our study advances policy research by showing that there are changing trends in the presence of school health policy topics and the strength of policy language in those content areas. Future research should examine how changes in trends are related to changes in school health environments and, subsequently, student health outcomes.

### 4.2. Future Directions for Research, Policy, and Practice

More schools are pushing for coordinated approaches to health that address many topic areas. As researchers, it is important that we identify how these topics compete for limited time and resources during the school day and identify approaches to improve health outcomes across multiple disciplines. This study suggests that most CIPs focus on at least two health topic areas; thus, multi-disciplinary interventions are likely needed. Our study also demonstrates regional- and district-specific differences in CIPs, suggesting that policymakers can encourage schools to adapt and tailor their CIPs based on students’ needs and unique local partnerships that can be leveraged to ensure effective implementation. For example, state agencies, such as the Texas Education Agency, can play a central role in creating more localized efforts or disseminating resources specific to the needs of various PHRs. 

An essential step that practitioners can take to strengthen their CIP is to ensure district improvement plans are synergistic with CIPs. They include comprehensive, specific, and strongly worded strategies that address the fundamentals of physical and emotional–social health students. For example, developing and using a checklist or template that includes all four health-related topics may be warranted. Furthermore, as nutrition and physical activity were the least included topic areas within CIPs, there may be opportunities to coordinate processes for developing and refining CIPs in coordination with other health policies to increase the inclusion and strength of nutrition and physical activity language within CIPs. For example, school districts’ health advisory councils could contribute to developing and evaluating CIPs, local wellness policies, and the use of comprehensive school health curricula. However, most of these policies are unfunded and unenforced mandates that are lower priorities for school districts.

## 5. Conclusions

School districts go beyond fulfilling students’ academic requirements by serving as a setting for addressing youths’ multi-faceted health needs and connecting families with resources that address nutrition, physical activity, mental health, and bullying. School CIPs help to signal these priorities, and most CIPs address two or more health topics. Further, there are important trends over time and by region, which can help to identify opportunities for interventions at the district level. These trends may also provide opportunities for tailoring CIPs at the school level. Schools alone cannot carry the sole responsibility for improving student health; thus, more robust and local tailoring of CIPs may be an opportunity for each school to leverage their existing partnerships with the community and for local programs to provide support to improve the health of children and their families [43].

## Figures and Tables

**Table 1:** Keywords Used to Search Campus Improvement Plans in Texas (SPAN 2015–2016 and 2019–2020).

	General Terms	Physical Activity	Nutrition	Mental Health	Bullying
Keywords	Health/y, wellness, BMI, well-being	Active, fitness, FitnessGram, physical education/ activity, recess	Food, meal, lunch, snack, diet, eating nutritious/al, fruit/vegetable, vending machine	Social health/skill, suicide, inclusion (specific to racial, ethnic, and/or gender inclusion), positive development, behavioral health, emotional/ly health/y, self-esteem, coping skills, emotional literacy, character education/development	Bullying, zero tolerance, violence

**Table 2:** Characteristics of Schools with Campus Improvement Plans in Texas (SPAN 2015–2016 and 2019–2020).

	PHR 1 (*n* = 34)	PHR 4/5N (*n* = 36)	PHR 6/5S (*n* = 49)	PHR 11 (*n* = 44)	*p*-Value	Total (*n* = 163)
Average Proportion African American/Black Students (SD)	4.6 (4.4)	21.4 (14.0)	15.1 (14.7)	0.9 (1.7)	<0.001	10.5 (13.3)
Average Proportion Hispanic Students (SD)	59.0 (23.5)	37.2 (21.8)	53.7 (27.9)	92.1 (13.0)	<0.001	61.5 (29.9)
Average Proportion White Students (SD)	31.8 (22.8)	36.8 (25.0)	22.4 (23.7)	5.9 (10.5)	<0.001	23.1 (24.0)
Average Proportion Asian Students (SD)	1.9 (2.3)	0.7 (0.9)	6.3 (9.9)	0.4 (1.1)	<0.001	2.6 (6.1)
Average Proportion American Indian or Pacific Islander Students (SD)	0.4 (0.4)	0.4 (0.4)	0.4 (0.4)	0.1 (0.2)	<0.001	0.3 (0.4)
Average Proportion of Students with Two or More Races/Ethnicities (SD)	2.3 (1.6)	3.5 (2.0)	2.1 (1.9)	0.5 (0.9)	<0.001	2.0 (2.0)
Average Proportion Economically Disadvantaged Students (SD)	67.7 (22.6)	74.7 (16.6)	60.3 (32.1)	85.5 (14.1)	<0.001	71.8 (24.8)
Average Proportion English Language Learning Students (SD)	14.2 (16.4)	25.3 (18.9)	29.8 (22.1)	35.1 (22.3)	<0.001	27.0 (21.5)
Average Proportion Special Education Students (SD)	9.8 (3.8)	10.1 (3.5)	7.9 (2.5)	10.2 (3.9)	0.005	9.4 (3.5)
Average Number of Students (SD)	489.4 (107.0)	532.2 (136.1)	788.7 (232.6)	579.4 (146.1)	<0.001	613.1 (205.8)
Average Campus Expenditures per Student in USD (SD)	6526.0 (873.3)	6517.4 (846.2)	6492.9 (1264.8)	7638.8 (1099.6)	<0.001	6814.5 (1165.3)
Urban–Rural Status					<0.001	
Major Urban	32.4%	2.8%	46.9%	0.0%		21.5%
Urban	26.5%	22.2%	40.8%	81.8%		44.8%
Rural	41.2%	75.0%	12.2%	18.2%		33.7%

Note: Demographic differences are due to regional variation in population demographic characteristics. PHR—Public Health Region. SD—Standard Deviation. USD—United States Dollars.

**Table 3:** CIPs (*n* = 163) including each topic area by Public Health Region (PHR).

	PHR 1 (*n* = 34)	PHR 4/5N (*n* = 36)	PHR 6/5S (*n* = 49)	PHR 11 (*n* = 44)
Physical Activity	52.9%	58.3%	63.3%	84.1%
Nutrition	32.4%	55.6%	61.2%	72.7%
Mental Health	55.9%	75.0%	75.5%	81.8%
Bullying	58.8% **(b)**	88.9% **(a)**	77.6%	75.0%
Total Number (Mean ± SD)	2.0 ± 1.5 **(c)**	2.8 ± 1.3	2.8 ± 1.3	3.1 ± 1.1 **(a)**

Notes: Statistical significance is based on the unadjusted models. In the adjusted models, the difference between the total number of topic areas addressed in PHRs 1 and 11 became marginally significant (*p* = 0.051). **(a)** = significantly different from PHR 1; **(b)** = significantly different from PHR 4/5N; **(c)** = significantly different from PHR 11.

**Table 4:** CIPs (*n* = 163) strength for each topic area by Public Health Region (PHR).

	PHR 1 (*n* = 34)	PHR 4/5N (*n* = 36)	PHR 6/5S (*n* = 49)	PHR 11 (*n* = 44)
Physical Activity (Mean ± SE)	1.5 ± 0.2	1.7 ± 0.2	1.6 ± 0.2	2.0 ± 0.2
Nutrition (Mean ± SE)	0.9 ± 0.4	0.8 ± 0.2 **(d)**	1.4 ± 0.3	1.6 ± 0.2 **(b)**
Mental Health (Mean ± SE)	1.4 ± 0.2	1.3 ± 0.2	1.8 ± 0.2	1.7 ± 0.2
Bullying (Mean ± SE)	1.8 ± 0.2	1.5 ± 0.1 **(c, d)**	1.9 ± 0.1 **(b)**	2.0 ± 0.1 **(b)**
Total Strength (Mean ± SE)	1.3 ± 0.3 **(d)**	1.7 ± 0.2	1.9 ± 0.2	2.1 ± 0.2 **(a)**

Notes: Statistical significance is based on the unadjusted models. **(a)** = significantly different from PHR 1; **(b)** = significantly different from PHR 4/5N; **(c)** = significantly different from PHR 6/5S; **(d)** = significantly different from PHR 11.

## Data Availability

The datasets analyzed during the current study are available from the corresponding author on request.
